# An MRI study of the tibial nerve in the ankle canal and its branches: a method of multiplanar reformation with 3D-FIESTA-C sequences

**DOI:** 10.1186/s12880-021-00582-8

**Published:** 2021-03-17

**Authors:** Yan Zhang, Xucheng He, Juan Li, Ju Ye, Wenjuan Han, Shanshan Zhou, Jianzhong Zhu, Guisheng Wang, Xiaoxia Chen

**Affiliations:** 1 Department of Radiology, Shandong Mental Health Center, Shandong, 250014 China; 2 Department of Radiology, Third Medical Centre of Chinese PLA General Hospital, Beijing, 100039 China; 3 Department of Urology, Third Medical Centre of Chinese PLA General Hospital, Beijing, 100039 China; 4 Department of Radiology, The Second Affiliated Hospital of Shandong First Medical University, Taian, 271000 China

**Keywords:** MR neuroimaging, 3D-FIESTA-C, Multiplanar reformation, Ankle canal, Tibial nerve and its branches

## Abstract

**Background:**

The visualization of the tibial nerve and its branches in the ankle canal is helpful for the diagnosis of local lesions and compression, and it is also useful for clinical observation and surgical planning. The aim of this study was to investigate the feasibility of three-dimensional dual-excitation balanced steady-state free precession sequence (3D-FIESTA-C) multiplanar reformation (MPR) display of the tibial nerve and its branches in the ankle canal.

**Methods:**

The subjects were 20 healthy volunteers (40 ankles), aged 22–50 years, with no history of ankle joint disease. The 3D-FIESTA-C sequence was used in the 3.0 T magnetic resonance equipment for imaging. During scanning, each foot was at an angle of 90° to the tibia. The tibial nerve of the ankle canal and its branches were displayed and measured at the same level through MPR.

**Results:**

Most of the tibial nerve bifurcation points were located in the ankle canal (57.5%), few bifurcation points (42.5%) were located at the proximal end of the ankle canal, and none of them were found away from the distal end. The bifurcation between the medial plantar nerve and the lateral plantar nerve was on the line between the tip of the medial malleolus and the calcaneus, and it’s angle ranged between 6° and 35°. In MPR images, the display rates of both the medial calcaneal nerve and the subcalcaneal nerve were 100%, and the starting point of the subcalcaneal nerve was always at the distal end of the starting point of the medial calcaneal nerve. In 55% of cases, there were more than two medial calcaneal nerve innervations.

**Conclusion:**

The 3D-FIESTA-C MPR can display the morphological features and positions of the tibial nerve and its branches and the bifurcation point’s projection position can be marked on the body surface. This method not only benefited the imaging diagnosis of the tibial nerve and branch-related lesions in the ankle canal, but it also provided a good imaging basis to plan a clinical operation of the ankle canal and avoid surgical injury.

## Background

The malleolus canal is a fibrous bony channel behind the medial malleolus, with the anterior wall being the distal tibia, the posterior wall being the posterior talus and calcaneus, and the flexor support band covering the surface. The anatomy of the tibial nerve and its major branches is largely determined by its position in the ankle canal [1]. The special anatomical structure and soft tissue space of the ankle canal make the ankle canal syndrome the most common nerve disease in this area, and its occurrence is often closely related to the nerve compression [2, 3]. For example, the incidence of ankle tunnel syndrome is 4–7% in China. This not only leads to tibial nerve dysfunction and plantar pain, but also mainly causes heel pain and even abductor atrophy of the little toe [4]. It is noteworthy that this body part is used as a pathway in minimally invasive and surgical operations [5]; ankle canal decompression, ankle canal incisions, and external nail fixation of fractures are likely to cause iatrogenic nerve injury [6–8]. Currently, through the anatomic study of the tibial nerve and its branches in the ankle canal, the location and course of the nerve are determined, and the origin and quantity of the medial calcaneus nerve and the inferior calcaneus nerve at the ankle canal are classified [9–11], which is useful for understanding the nerve in the ankle canal. Using ultrasound to display the nerves in this area and injection into the infracalcaneus nerve under the guidance of ultrasound can improve the injection accuracy [12, 13], but ultrasound cannot display the whole shape of the nerves in a stereoscopic and intuitive way, and it largely depends on the technology and experience of the operator. Some studies were performed to initially discuss the value of MR in the diagnosis and display of the tibial nerve and its branches in the ankle canal [14, 15]. Due to the direction limit and very thick slices of the 2D sequence, it is difficult to display the morphology of the nerve completely in the same plane, and it is difficult to display the small branches clearly. This study applied the three-dimensional double excitation balanced steady-state free precession sequence (3D-FIESTA-C) multiplanar reformation method to show the direction, position, and branches of the nerve in the vertical axis direction according to the neural anatomy, and describe the origin of the different branches and position change. This benefited the MR diagnosis of peripheral neuropathy of the ankle tube, and it also directly guided the clinical observation and surgical planning in image anatomy. The position of the nerve is successfully projected on the body surface through a certain measurement method, which is necessary to ensure the safety of the operation around the ankle canal.

## Methods

In our study, we used 40 ankle MRI images from 20 volunteers with an average age of 33.8 years (range from 22 to 50 years) who agreed to participate in the study and completed the informed consent form. They had no history of ankle disease and pain.

### MR scan parameter

We used HD 8 channels foot ankle coil from General Electric company, and the patient was placed in the supine position, with the toe pointing vertically up and the foot at an angle of 90° to the tibia. The patients’ ankle was placed into the coil horizontally and fixed. See Table 1 for specific scanning parameters of the 3D-FIESTA-C sequence.

### Image reformation method

**Fig. 1:**
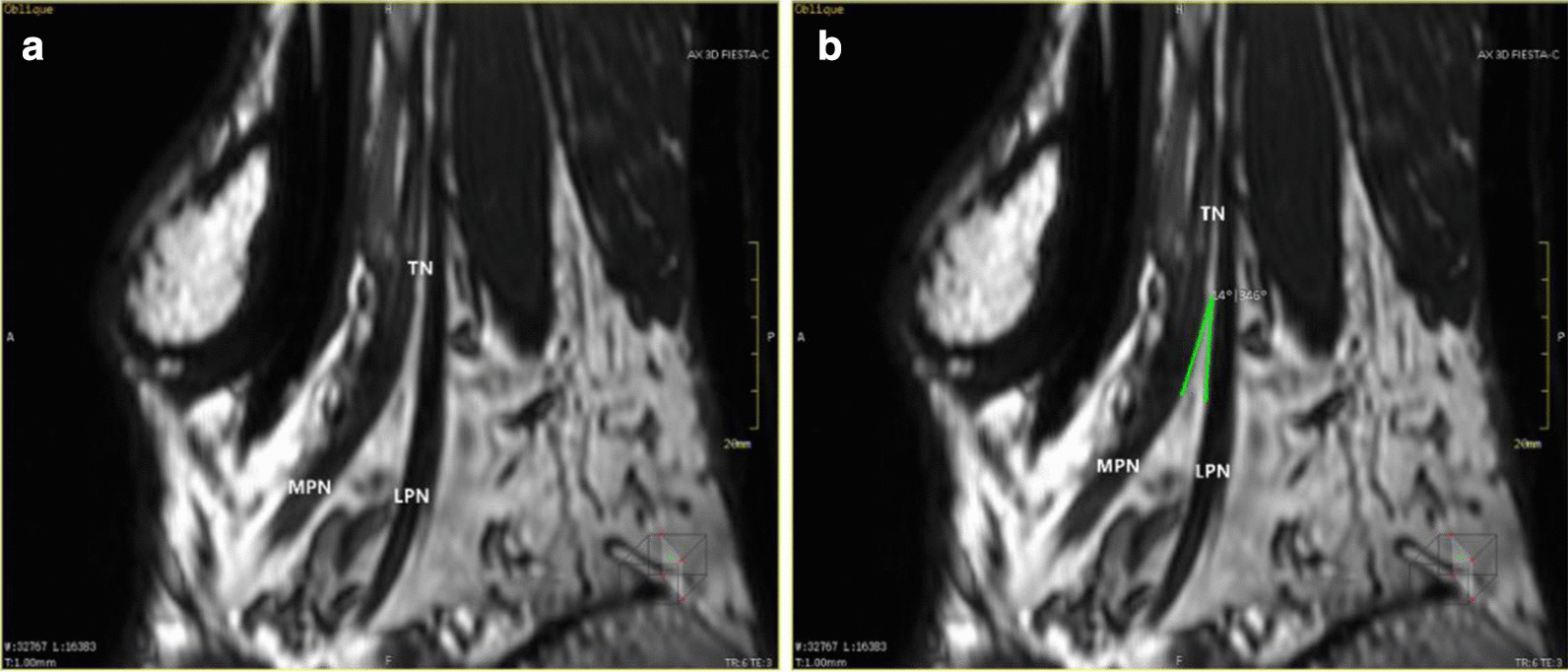
MPR shows the bifurcation of the medial and lateral plantar nerves at the same plane and bifurcation angle measurement: **a** the image of the oblique sagittal plane shows the bifurcation of the tibial nerve, the medial and lateral nerves of the plantar; **b** the angle between the medial and lateral nerves of the plantar can be measured on the oblique plane passing through the bifurcation of the tibial nerve

**Fig. 2:**
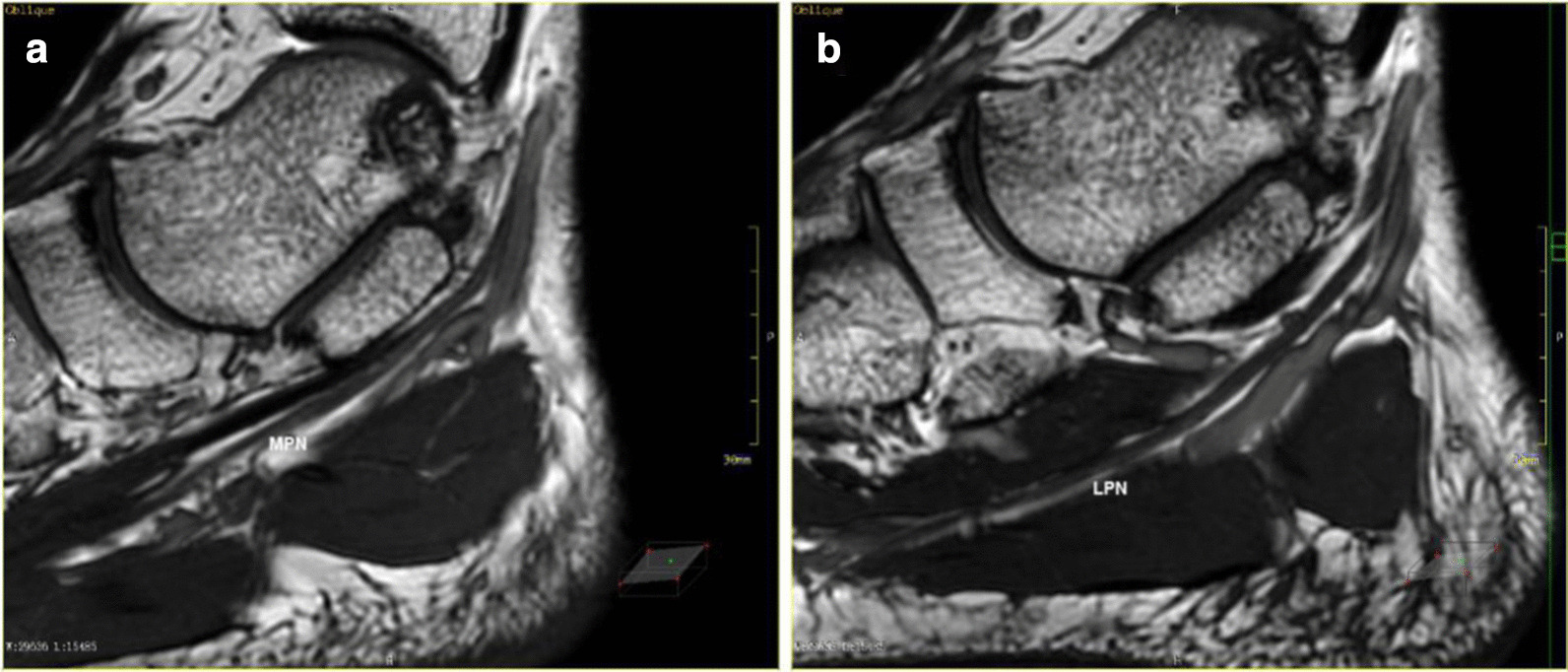
MPR shows images of medial and lateral plantar nerve: **a** the image of the oblique coronal plane that shows the medial plantar nerve in maximum range; **b** the image of the oblique coronal plane that shows the lateral plantar nerve in maximum range

**Fig. 3:**
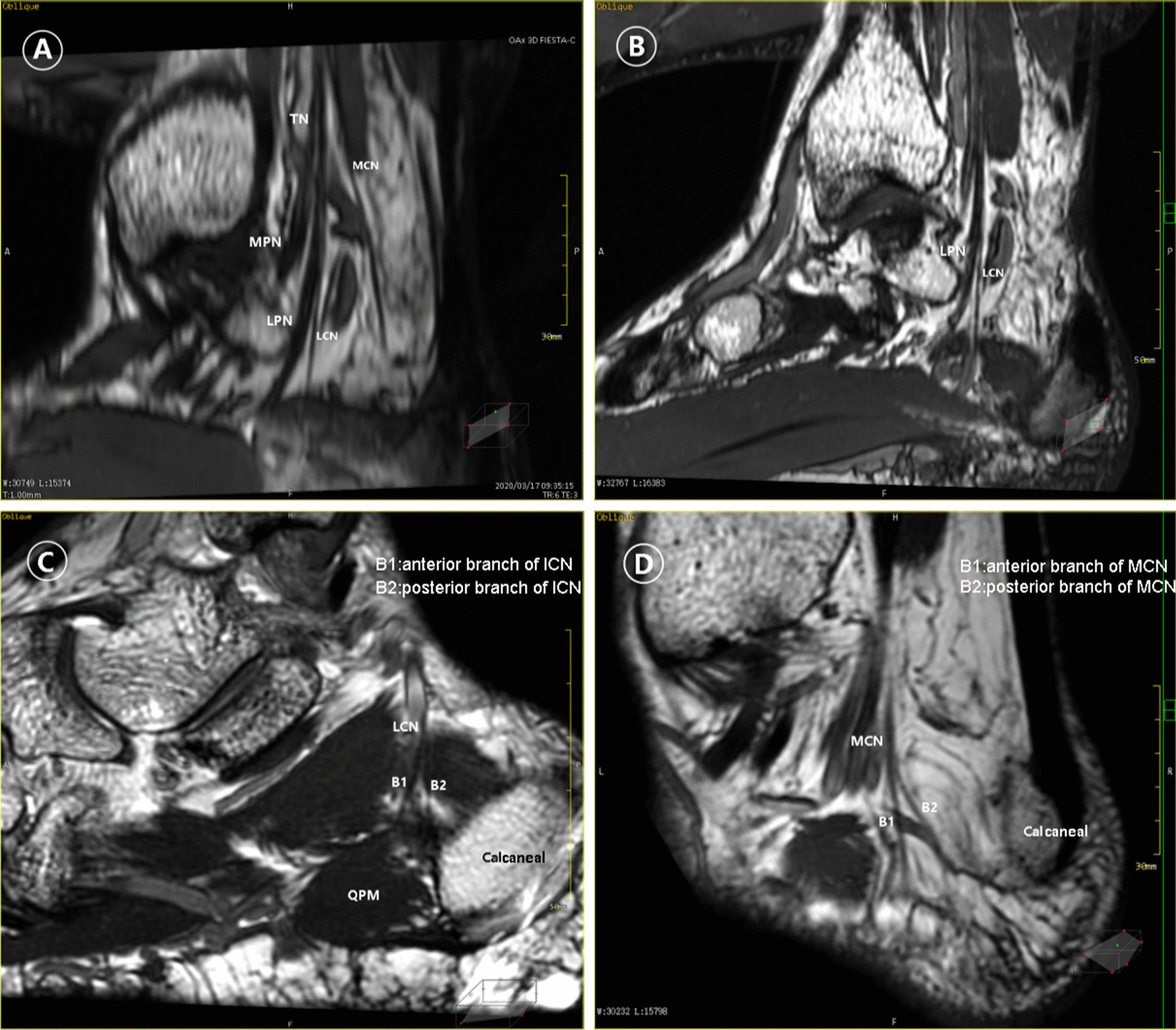
MPR shows the medial calcaneal nerve and the inferior calcaneal nerve: **a** the maximum extent of the medial calcaneal nerve is displayed on the oblique sagittal plane at the bifurcation of the medial calcaneal nerve; **b** the inferior calcaneal nerve is displayed on the oblique sagittal plane at the bifurcation of the inferior calcaneal nerve, which is emitted from the lateral plantar nerve; **c** the end of the inferior calcaneal nerve shown by the segmented reformation; **d** Segmental reformation of the medial calcaneal nerve ends

**Fig. 4:**
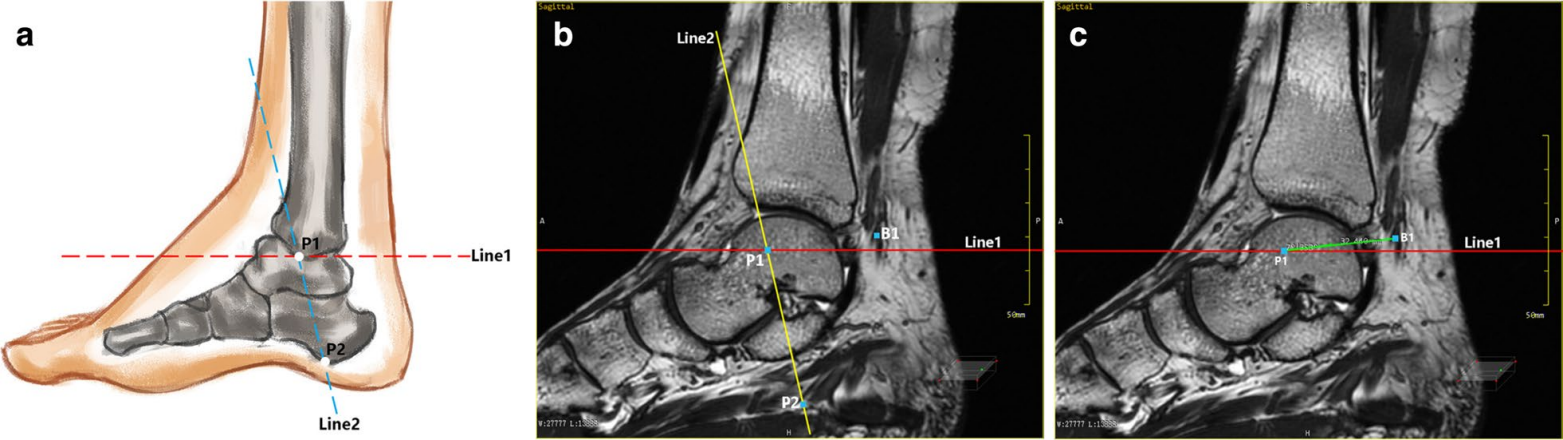
The relative position of the ankle canal reference line l and the medial and lateral nerve: **a** a schematic diagram showing two projection lines,P1 is projection of the lowest point of the medial malleolus and P2 is projection of the calcaneal tuberosity on the sagittal image; **b** the relative position of the bifurcation point and Line 1 and Line 2 on the sagittal image through the bifurcation point of the medial and lateral nerves of the plantar; **c** measure the distance between the bifurcation point and P1 and the angle between the bifurcation point and Line 1 on the sagittal image passing through the bifurcation points of the plantar and lateral nerves

**Fig. 5:**
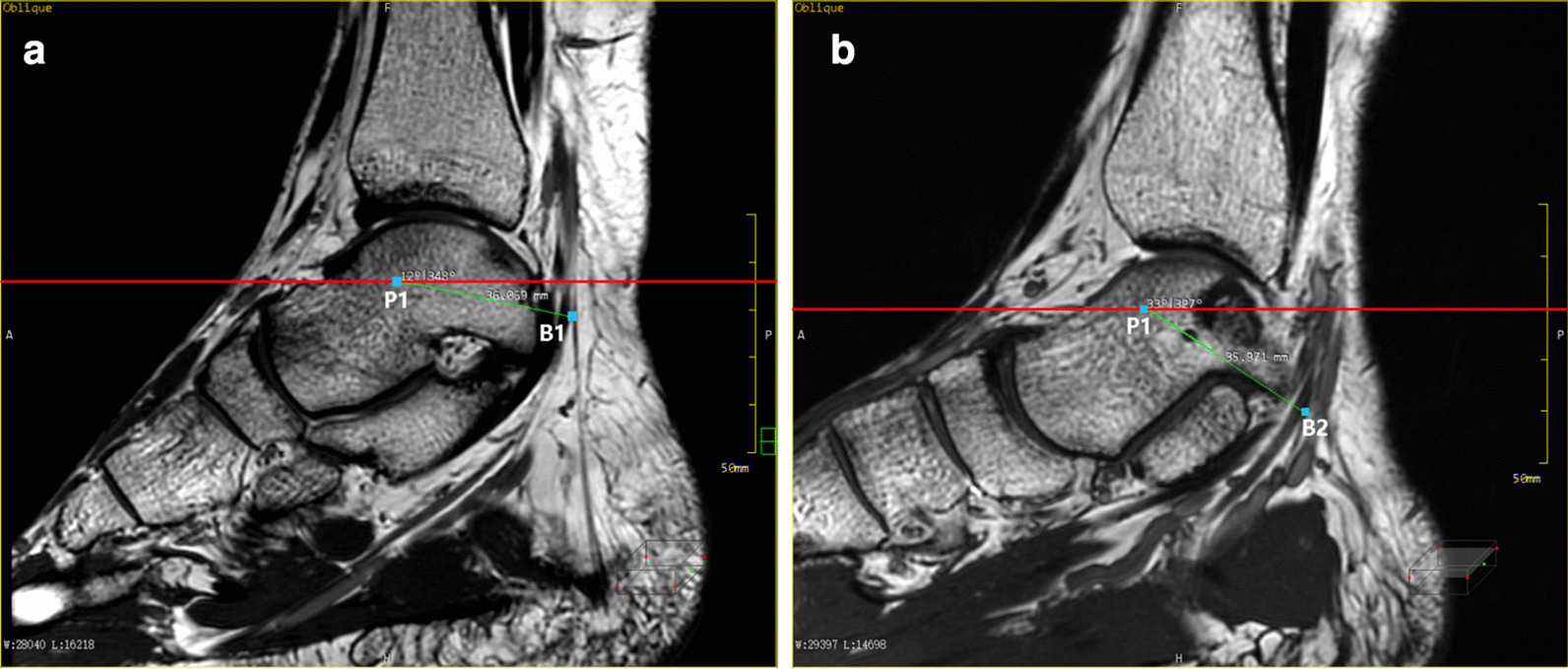
Measurement of the relative position of the medial and inferior calcaneal nerve on the sagittal plane: **a** measure the distance between the bifurcation point of MCN (B1) and projection of the lowest point of the medial malleolus (P1) and the angle between the bifurcation point and Line1; **b** the distance between the bifurcation point of ICN (B2) and P1, and the angle between the bifurcation point and Line 1 are measured

The images were loaded into the post-processing workstation and the MPR mode was selected. The specific reformation method was as follows: (1) The tibial nerve was identified on the cross-sectional images, and it was set as the center of rotation. Through slow, constant rotation, MPR increased the tibial display length to show the bifurcation between the medial plantar nerve and the lateral plantar nerve, as shown in Fig. 1a. Then, the center of rotation was placed at the bifurcation point, and the tibial nerve, the medial plantar nerve, and the lateral plantar nerve were shown in the same plane through appropriate small rotation. The wide window was appropriately adjusted to improve the resolution and contrast of the nerve, and the angle between the medial plantar nerve and the lateral plantar nerve was measured, as shown in Fig. 1b. (2) The largest image was reformatted through the medial plantar nerve and the lateral plantar nerve, as shown in Fig. 2. (3) The tibial nerve, the lateral plantar nerve, and the longitudinal axis of the medial plantar nerve were considered as the rotation axis, and they were rotated to see if there were any other branches. If there are branches, the center of rotation was placed on the bifurcation and the branches were displayed at the maximum level, as shown in Figs. 3a and 4b. Further segmental reformation was carried out to the nerve terminal to determine its dominant region. Meanwhile, the accuracy of the reformatted image of the nerve was checked against the cross-sectional image as shown in Fig. 3c, d. (4) Two reference lines were set on the sagittal plane image: the projection of the horizontal line of the medial malleolus on this plane (Line 1) and the projection of the medial malleolus and calcaneus nodules on this plane (Line 2). The relationship between the bifurcation points and Line 1 and Line 2 was determined on the sagittal plane images of the bifurcation points of the inner and outer plantar nerves. The distance between the bifurcation point and the projection point of the medial ankle tip was measured on the plane and the included Angle between the bifurcation point and the projection point of the medial ankle tip and Line 1 was measured, as shown in Fig. 4. For the convenience of marking, the angle of the bifurcation point above the horizontal line was positive, and the angle below the horizontal line was negative. Then, the distance from the bifurcation point to the projection point of the lateral ankle tip on the plane was sequentially measured on the sagittal plane image of the bifurcation point passing through the other branches, as shown in Fig. 5. For the convenience of marking, the angle of the bifurcation point above the horizontal line was positive, and the angle below the horizontal line was negative.

## Results

**Fig. 6:**
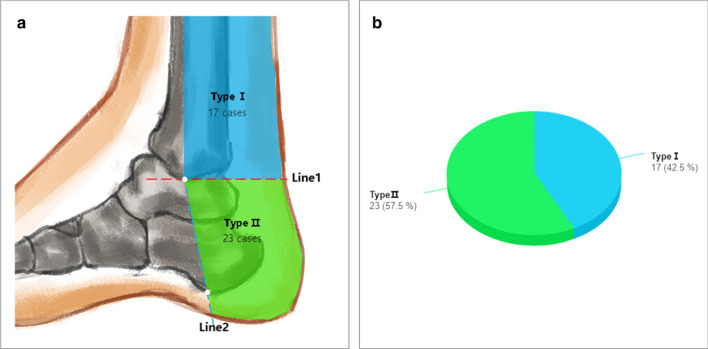
Types of the location of the bifurcation of the medial and lateral plantar nerves: **a** location illustration of the bifurcation points of the medial and lateral plantar nerves at the ankle canal. **b** Scale diagram of the location of the bifurcation points of the medial and lateral plantar nerves

All the images (40/40) clearly showed the tibial nerve (TN) at the ankle canal and the bifurcation of the medial and lateral plantar nerves (MPN and LPN), with the gap of fat, the posterior tibial artery and vein besides the bifurcation. According to the position relationship between the medial and lateral plantar nerve bifurcation points, the projection of the horizontal line passed through the lowest point of the tip of the medial malleolus on the sagittal image (Line 1), and the projection of the line was connected to the tip of the medial malleolus and the calcaneus nodules on the sagittal image (Line 2). The result can be divided into three types. Type I: 17 out of 40 cases (42.5%) showed an ankle tube proximal tibial bifurcation point that was located at Line 1. Type II: the bifurcation point was located between Line 1 and 2 in 23 cases (57.5%). Type III: the bifurcation point was located far on Line 2, and we did not find this type of image. All of the above descriptions are shown in Fig. 6.

**Fig. 7:**
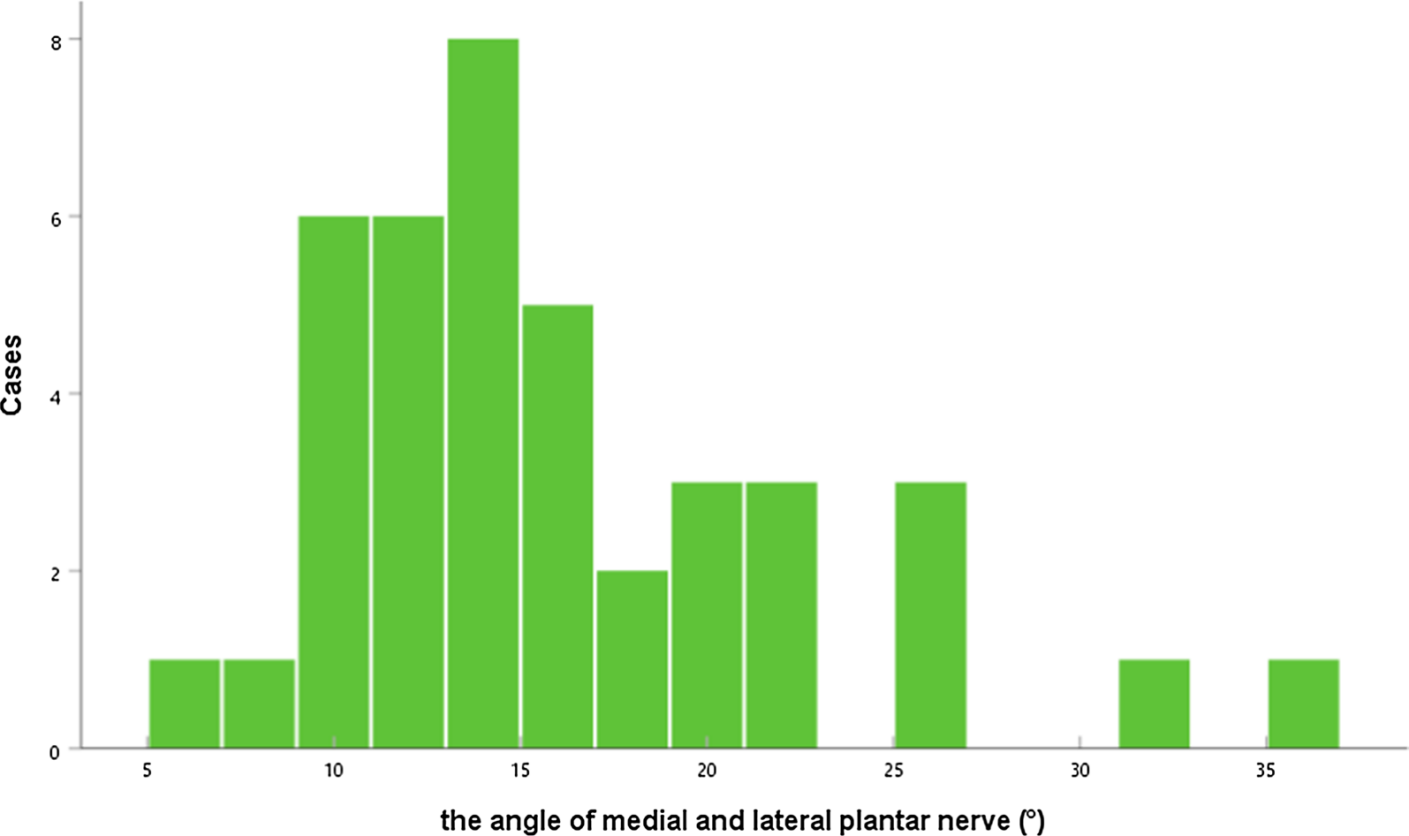
The angle between the medial and lateral plantar nerves ranged from 6° to 35° and the overall level was 14 (12,19.8)°. The figure show the specific distribution frequency histogram

The reformation image of the medial and lateral plantar nerve was uniform in thickness and tapering from near to far, and the display range was obviously larger than that of the two at the same time, but when the two were displayed at the same time, the bifurcation position and morphology could be defined. The angle of the medial and lateral plantar nerves ranged between 6° and 35°, as shown in Fig. 7.

**Fig. 8:**
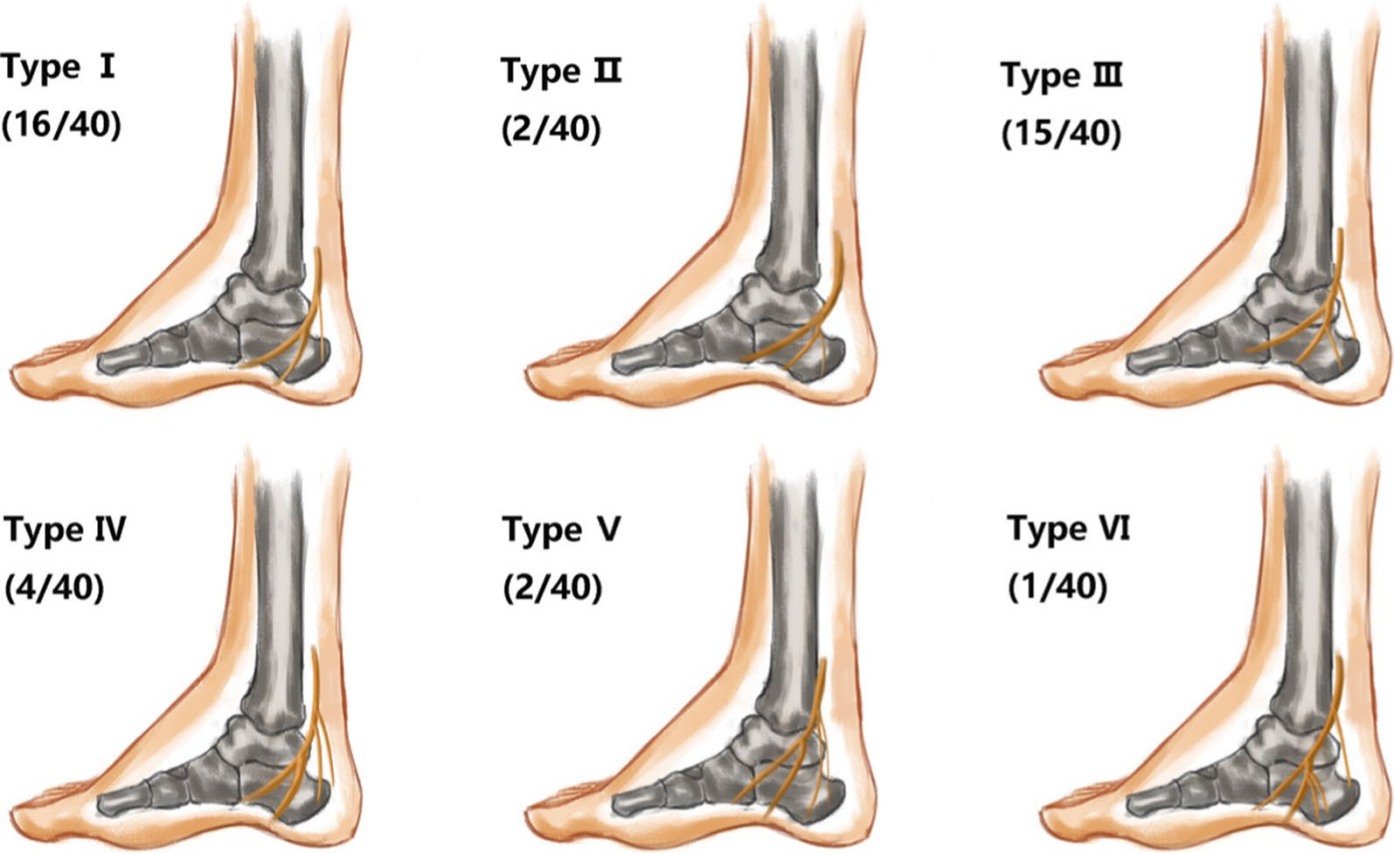
Modes of origins of the medial calcaneal nerve: Type I a medial calcaneal nerve originating from the tibial nerve; Type II a medial calcaneal nerve originating from the lateral plantar nerve; Type III two medial calcaneal nerves, one originated from the tibial nerve, and the other originated from lateral plantar nerve; Type IV two medial calcaneal nerves, both originated from tibial nerve; Type V two medial calcaneal nerves, with a common origin on the tibial nerve; Type VI three medial calcaneal nerves, one originated from the tibial nerve and the other two originated from the lateral plantar nerve

**Fig. 9:**
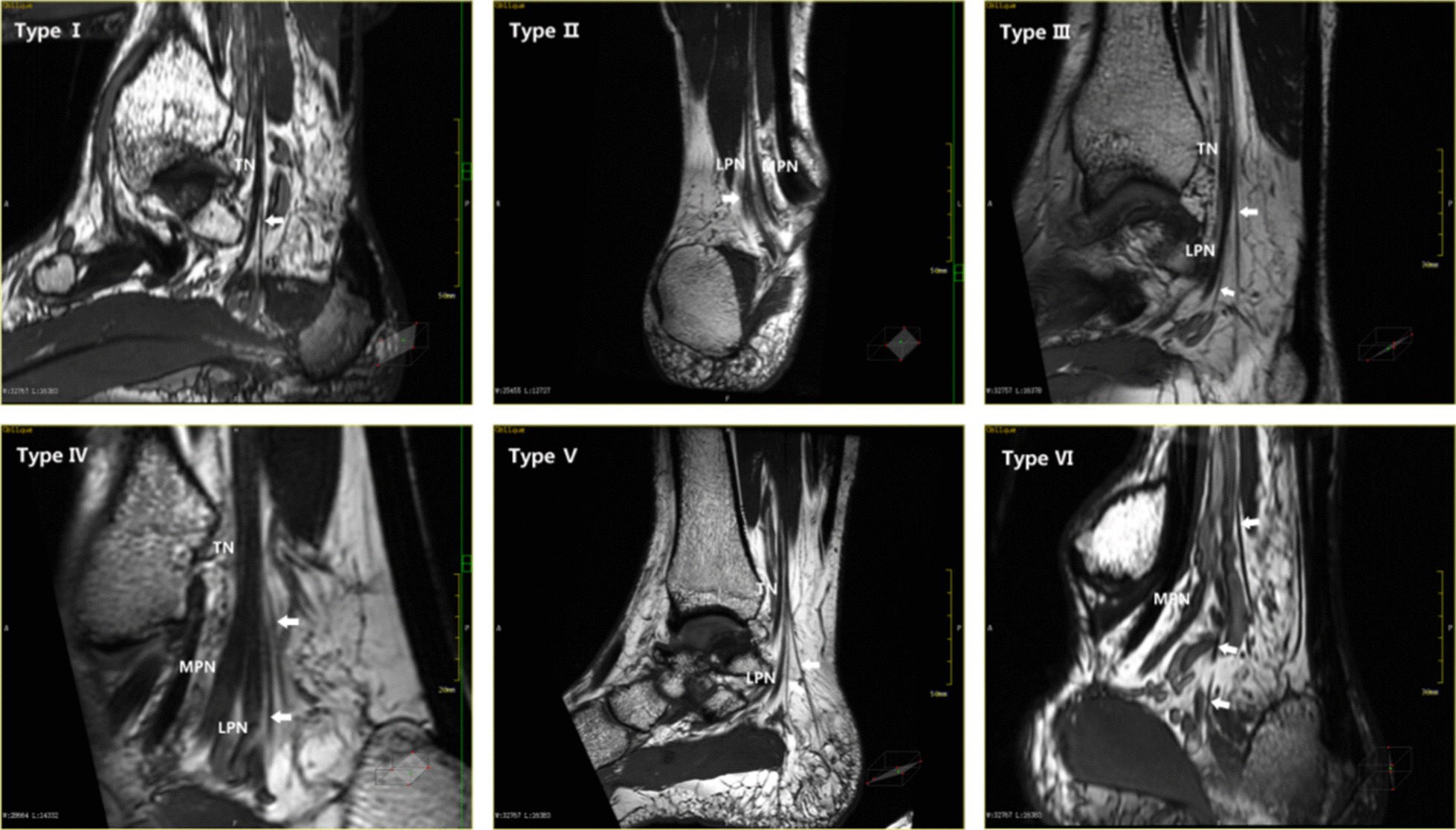
Reconstructed image of the medial nerve branch of the calcaneus (white arrow): MPR images of 6 types of the medial calcaneal nerve branches. Because the three nerves of type VI are not in the same plane, the MPR image cannot display their bifurcation points at the same plane

The occurrence rate of the medial calcaneal nerve (MCN) was 100%, although the number and starting position of the nerve varied greatly. Segmental reformation showed that the distal part of the nerve was located behind the calcaneal tuberosity and the subcutaneous tissue of the heel. Out of the 40 images in our study, 18 had a single medial calcaneal nerve, 21 had two medial calcaneal nerves, and only one had three medial calcaneal nerves. From the position of origin, the medial calcaneal nerve started from the tibial nerve, the plantar nerve, the lateral nerve bifurcation point, and the lateral plantar nerve. A medial calcaneal nerve originating from the medial plantar nerve was not found. Based on the MPR data, we divided the medial calcaneal nerve into major types, as shown in Figs. 8 and 9.

**Fig. 10:**
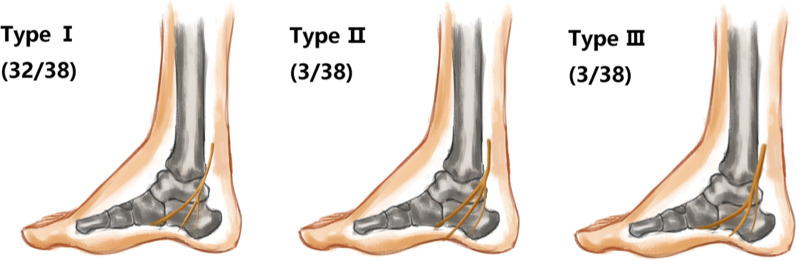
Modes of origins of the inferior calcaneal nerve: Type I the inferior calcaneal nerve originates from the tibial nerve; Type II the inferior calcaneal nerve originates from the bifurcation of the lateral plantar nerve; Type III the inferior calcaneal nerve originates from the tibial nerve

**Fig. 11:**
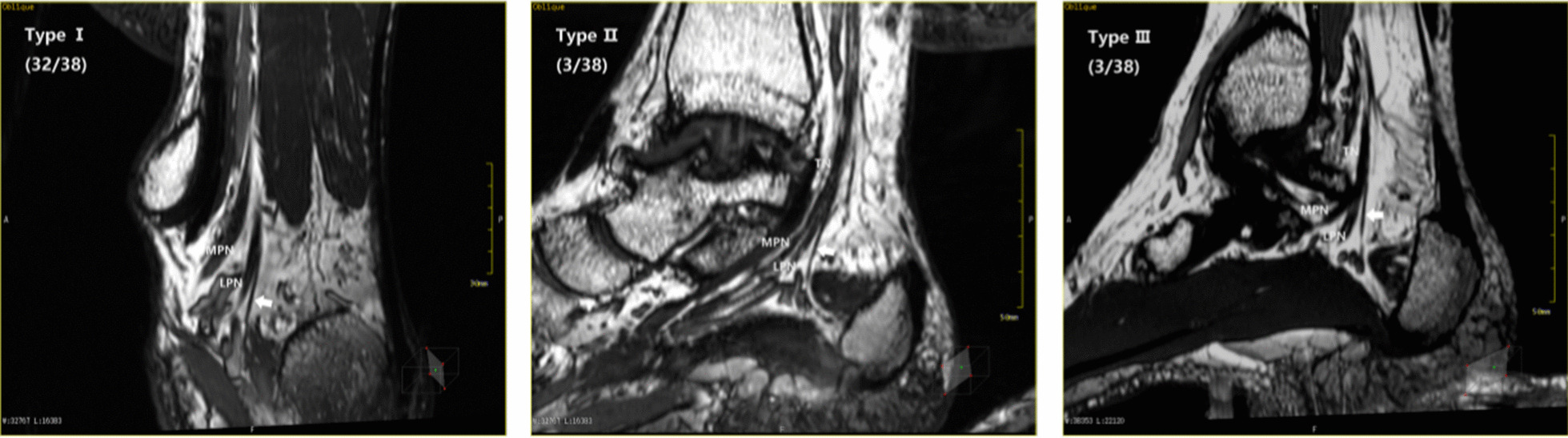
Reconstructed image of the inferior calcaneal nerve branches (white arrow): MPR images of 3 types of the inferior calcaneal nerve branches

The inferior calcaneal nerve (ICN) was identified in 38 out of 40 cases. In 32 cases, the inferior calcaneal nerve originated from the lateral plantar nerve. In three cases, the inferior calcaneal nerve originated from the bifurcation of the medial and lateral plantar nerves. In three cases, the inferior calcaneal nerve originated from the tibial nerve. The origin locations are mainly shown in Figs. 10 and 11. In this group of images, one subcalcaneal nerve was reconstructed, and no more than two medial calcaneal nerve types were found. The starting point of the inferior calcaneal nerve was always located at the distal end of the starting point of the medial calcaneal nerve, and its endings were distributed in front of the calcaneal tuberosity and the abductor of the little toe, as shown in Fig. 3c.

**Fig. 12:**
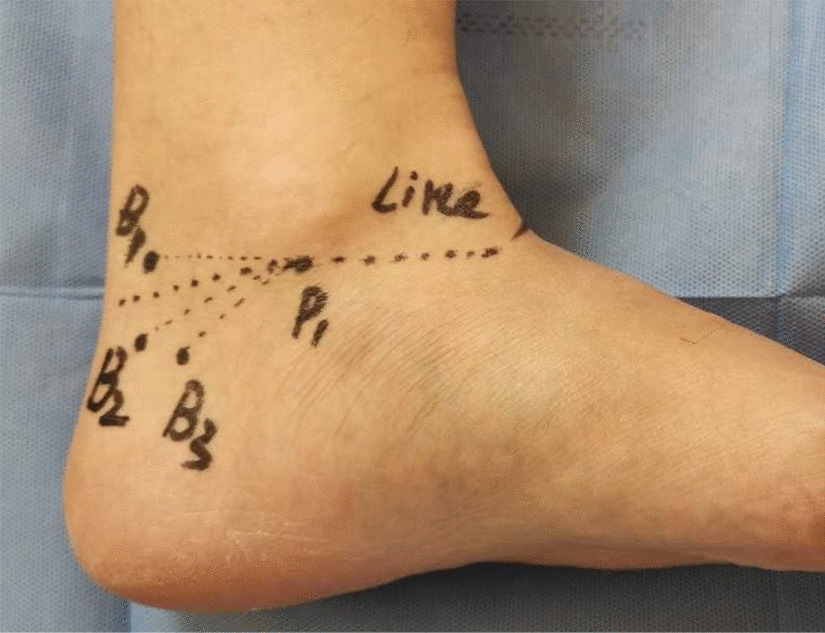
Projection point of the bifurcation point on the surface of the medial malleolus: The projection points of the projection of the bifurcation point of the medial and lateral nerves of the plantar (B1), the projection of the starting point of the medial calcaneal nerve (B2), the projection of the starting point of the inferior calcaneal nerve (B3) and the horizontal line passing through the lowest point of the tip of the medial malleolus (Line) can be marked on the surface of the inner ankle by measuring their positions

By measuring the distance to the projection point of the medial malleolus tip on the sagittal position of different bifurcation points and the included angle between the two lines and the projection on the plane through the horizontal line of the medial malleolus tip, each point can be marked inside of the ankle joint, as shown in Fig. 12.

## Discussion

The ankle canal is a narrow fibrous bone channel in anatomy, including the tibial nerve and its branches, posterior tibial blood vessels, and deep flexor tendon of the calf. The nerve channels in the ankle canal are divided into four septa by the fascia, and branches of the tibial nerve travel at different intervals [16]. These anatomical structures in the ankle canal are often related to many diseases, and the fine anatomy of the nerve in this area can be shown morphologically by MR, which is of great significance for the diagnosis and clinical treatment of related diseases [15, 17].

### Characteristics of tibial nerve and its branches on 3d-FIESTA-C sequence

Currently, the MR research on peripheral nerve imaging mainly focuses on diffusion tensor imaging (DTI). However, DTI is susceptible to many factors of magnetic field and spatial and contrast resolution, and it lacks the accuracy for evaluating small branches [18]; hence, it is mainly applied to large nerves and branches. Further studies are needed to determine its role in daily clinical practice [19]. There are few studies on the nerve morphology of the tibial nerve in the ankle canal with other MR sequences. In one study, Farooki et al. [14] preliminarily showed the inner and outer calcaneus nerves by using the orthogonal plane to conduct thin layer scan on the corpse, while Donovan et al. [15] proposed that the inner and outer calcaneus nerves were more obvious in the oblique coronal plane. As the 3D-FIESTA-C sequence is encoded in the 3D space, the inter-layer resolution of the 3D sequence is very high. A thin layer can ensure high-quality reformation images on any plane, and it can clearly show relatively small nerve branches. Hatipoglu et al. [20] applied this sequence to the study of posterior fossa nerve imaging. In the images of the 3D-FIESTA-C sequence, the nerve showed low signal, and between the muscle (slightly lower signal) and the tendon (lower signal), the peripheral fat showed high signal; the blood vessels showed high signal and the thicker blood vessels showed a low signal clipping sign. These are necessary conditions to show clear and distinguishable neural structures in multi-plane images. On the image of the transection of the tibial nerve, although the tibial nerve presents low signal on the whole, multiple low-signal nerve fiber bundles and slightly higher signal intervals between the fiber bundles can be seen inside, and fat high signal can be seen around the tibial nerve [21]. The accompanying posterior tibial artery and vein on the medial side of the nerve should be noted. Because the tibial nerve and its branches are striped structures and a main longitudinal axis direction line along the human body, most of the nerves showed a wide range of morphology through oblique sagittal plane or oblique coronal plane. A few nerves that cannot be completely depicted in one plane can be reformatted to 2–3 planes to show their shape, and these planes will not affect the judgment of nervous (Fig. 3a–d).

### The bifurcation position and branching pattern of the tibial nerve and branches at the ankle canal

The tibial nerve is generally cylindrical running behind and below the medial malleolus in the ankle canal, with two main branches: the medial plantar nerve and the lateral plantar nerve. Of course, Develi [22] reported a unique case of three branches, but this is very rare. The location of the distal branch of the tibial nerve is not constant. Bareither et al. [23] pointed out that the branch point can be within the range from 2.8 cm from the distal end of the medial malleolus tip to 14.3 cm from the proximal end. Dellon et al. [24] reported that the bifurcation point was within 2 cm of the malleolar-calcaneal axis. By analyzing 50 cases, Torres et al. [9] found that 88% of them were located in the ankle canal and 12% were located in the proximal end of the ankle canal. In this study, the medial plantar nerve and the lateral plantar nerve were divided into three types through positioning of the branch point, and 42.5% of the bifurcation points were located in the proximal part of the ankle canal (type I); 57.5% of the bifurcation points were located in the ankle canal (type II), and no bifurcation point was found far from the distal end of the ankle cannal (type III). These results were similar to those reported by Torres. In this study, the angle measured between the inner and outer plantar nerves ranged between 6° and 35°. The medial plantar nerve is one of the larger branches of the tibial nerve. It is located on the lateral side of the posterior tibial artery and in front of the medial plantar artery [17, 25]. The lateral plantar nerve is a smaller branch that runs between the inner and lateral plantar arteries [17]. The purpose of reformatting a single branch of the inner and outer nerves of the plantar sole and displaying them at the maximum display level is to clearly observe their morphology, their course, and whether there is compression on the pathway.

The medial calcaneal nerve is one of the main branches of the tibial nerve, terminating in the skin of the heel and the weight-bearing surface, and providing sensory innervation to the inner posterior side of the heel [26, 27]. The starting position and the number of medial calcaneal nerves vary greatly. Quantitatively, Dellon et al. [28] found that 37% had one medial calcaneal nerve, 41% had two medial calcancal nerves, 19% had three medial calcancal nerves, and 3% had four medial calcancal nerves. Kim et al. [29] and Yang et al. [10] found that there were up to five medial calcaneal nerves. Govsa et al. [11] and Kim et al. [29] indicated that there were two common vessels on the medial surface of the calcaneus. In this study, up to three medial calcaneal nerves were reformatted, which may be related to the small number of samples. However, a maximum of two medial calcaneal nerves were reformatted in this study, which is consistent with the above-mentioned views. At the starting point, the medial calcaneal nerve may originate from the tibial nerve, the medial and lateral plantar nerve bifurcation points, and the lateral plantar nerve. Havel et al. [30] and Dellon et al. [28] found that the medial calcaneal nerve can also originate from the medial plantar nerve. These differences are mainly related to more than one calcaneal nerve in most cases, indicating the high rate of variation in the origin of the medial calcaneal nerve. Although the medial calcaneal nerve can originate from the tibial nerve to the lateral plantar nerve, segmental reformation showed that its terminal branches showed a consistent range of innervation, all of which went to the heel skin behind the calcaneal tuberosity, which was consistent with anatomic conclusions [10, 11, 29, 31].

The inferior calcaneal nerve is also known as the first lateral plantar nerve, the little toe abductor nerve, or the Baxter nerve [32–34]. Moroni et al. [13], Oliva et al. [35] and Govsa et al. [11] found that the occurrence rate of this nerve was 100%. In our reconstructed image, the display rate was 95% and the anatomical data showed that the cross-sectional diameter at the beginning of the subcalcaneal nerve was 1.4 ± 0.5 mm. It is possible that the cases in which the nerves were not clearly shown are related to the fine nerve, but this requires further study. The inferior calcaneal nerve always appears as a single branch, which was confirmed in our study, and no more than two medial calcaneal nerves were found. The origin position of the inferior calcaneal nerve is not constant. Arenson [36], Didia [31], and Govsa [11] et al. believed that the inferior calcaneal nerve could originate from the lateral plantar nerve, the medial plantar nerve, the lateral nerve bifurcation, and the tibial nerve, but our research results are consistent with those of Louisia et al. [37] and Kim et al. [38]. In all images, most of the inferior calcaneal nerves originated from the lateral plantar nerve. In this study, we also found that the subcalcaneal nerve terminal shown by segmental reformation often disappeared in the muscular space or surrounding area in front of the tibial tubercle, and occasionally two branches could be reconstructed, with the anterior branch distributed in the area of the abductor muscle of the little toe and the posterior branch distributed in front of the calcaneal tubercle.

### Clinical significance of positioning nerve bifurcation on body surface

Ankle canal lesions, especially ankle canal syndrome, often requires minimally invasive interventional therapy or even surgical treatment [39]. During the surgical incision and route planning, positioning the nerve can avoid the nerve damage to the greatest extent, so that the patients can experience better treatment effect and less complications. When using the external fixation of the fracture, positioning the nerve can also minimize the nerve damage. During nerve block, the effective injection site of ankle canal for heel pain can be determined by positioning the nerve [40]. In this study, we considered the horizontal line passing through the medial malleolus tip as the reference line, and by measuring the distance from the bifurcation point to the medial malleolus tip and the included angle with the reference line, we could conveniently mark the body surface registration point of each point on the medial malleolus. This method can also be used to localize the lesion in the body surface and measure its depth by the coronal position. These factors are of great significance for the invasive treatment of feet and ankles.

## Conclusion

By applying MPR on the 3D-FIESTA-C sequence, the morphological description and classification of the nerve structure in the ankle canal were carried out in detail. By measuring the distance between each bifurcation point and the tip of the medial malleolus, and the angle between the line and the horizontal line passing the tip of the medial malleolus, the projection position of the bifurcation point on the body surface was marked. This method not only benefited the imaging diagnosis of the tibial nerve and branch-related lesions in the ankle canal, but it also provided a good imaging basis to plan the clinical operation of the ankle canal and avoid surgical injury.

**Table 1:** Acquired parameters of the 3D-FIESTA-C sequence

Acquired the parameters	The value
Scan plane	Oblique (3 d)
Slice thickness	1.0
The TR	6.4
TE	3.0
Slabs	1
The flip angle	60
Frequency	288
Phase	288
NEX	2.00
Bandwidth	62.50
Matrix size	512 × 512
The SAR	3.0
The time of acquisition	6 min 40 s–7 min 28 s

## Data Availability

The datasets generated and/or analysed during the current study are available from the corresponding author on reasonable request.

## Abbreviations

3D-FIESTA-C: Three-dimensional dual-excitation balanced steady-state free precession sequence
MPR: Multiplanar reformation
TN: Tibial nerve
MPN: Medial plantar nerve
LPN: Lateral plantar nerve
MCN: Medial calcaneal nerve
LCN: Inferior calcaneal nerve
QPM: Square plantar muscle
